# Metagenomic next-generation sequencing reveals cross-reactivity of lateral flow cryptococcal antigen assay with *Trichosporon inkin*

**DOI:** 10.1128/asmcr.00140-25

**Published:** 2025-11-04

**Authors:** Eileen M. Burd

**Affiliations:** 1 Department of Pathology and Laboratory Medicine, Emory University School of Medicine 12239, Atlanta, Georgia, USA; 2 Department of Medicine, Division of Infectious Diseases, Emory University School of Medicine 12239, Atlanta, Georgia, USA; Vanderbilt University Medical Center, Nashville, Tennessee, USA

**Keywords:** next-generation sequencing, metagenomic next-generation sequencing, cryptococcal antigen, fungal meningitis, cross-reactivity

## Abstract

Metagenomic next-generation sequencing (mNGS) in plasma, cerebrospinal fluid (CSF), and bronchoalveolar lavage fluid is a relatively new technology that offers a means to potentially provide a diagnosis in cases where infection is suspected, but conventional diagnostic testing has not revealed a pathogen. There have been many publications of individual cases and overall appraisals of its utility in detecting bacteria, fungi, and DNA viruses associated with otherwise undiagnosed systemic infections. A recent article by Phillips et al. published in *ASM Case Reports* (2:e00053-25, 2025, https://doi.org/10.1128/asmcr.00053-25) presents a case of meningitis in an immunosuppressed child that was ultimately determined to be caused by *Trichosporon inkin* using mNGS. Elevated ß−1,3-D-glucan (BDG) levels in CSF and serum projected a diagnosis of fungal meningitis. Bacterial, fungal, and mycobacterial cultures were negative. Positive lateral flow cryptococcal antigen titers in serum and CSF complicated the anticipated diagnosis since *Cryptococcus* spp. are thought to not have sufficient cell wall BDG to produce positive test results. Given the ultimate diagnosis of *T. inkin* meningitis and the known cross-reactivity with *Trichosporon asahii* per package insert, the unexpected cryptococcal antigen results raised the possibility of additional cross-reactivity. The authors uncovered this possibility by testing three known clinical isolates of *T. inkin* which generated positive results. This case adds to the growing literature that highlights the utility of mNGS in providing a diagnosis in otherwise unresolved cases and shows that mNGS can be further instructive in elucidating limitations in commonly used diagnostic tests.

## COMMENTARY

The process of diagnosis can be complex, and there are many reasons for diagnostic uncertainty in infectious diseases, including atypical presentation, overlapping symptoms of many bacterial, viral, and non-infectious conditions, inadequate or inconclusive diagnostic tests, decreased test sensitivity in patients who have received antimicrobial agents, rare or unexpected pathogens, etc. Patients who have infections without a definitive diagnosis could suffer consequences if the ability of providers to administer the most effective targeted treatment is hindered or delayed. A recent article by Phillips et al. (1) published in *ASM Case Reports* presents a case of *Trichosporon inkin* meningitis in an immunosuppressed child that was diagnosed using metagenomic next-generation sequencing (mNGS) and uncovered previously undescribed cross-reactivity with cryptococcal antigen lateral flow tests.

Newer innovative diagnostic methods such as NGS can facilitate diagnosis when traditional microbiologic approaches fail to identify a pathogen (Table 1). One type of NGS directly from patient samples is broad-range PCR amplification of differing targets (bacterial, fungal, and/or mycobacterial depending on the type of infection suspected) followed by sequencing (University of Washington Molecular Diagnosis Microbiology Section, Seattle, WA, USA, Mayo Clinic Laboratories, Rochester, MN, USA). Another type of NGS is mNGS. This is a target-agnostic approach that does not require prior suspicion of a target pathogen(s) and can be helpful in making diagnoses especially when collection of a more invasive sample from the primary infection is not practical. The Karius Test (Karius Inc., Redwood City, CA, USA) (2–7) and Delve Detect CSF (Delve Bio, Inc., San Francisco, CA, USA) (8–13) are mNGS tests that have become instrumental in the diagnosis of infectious disease in many complex cases.

**TABLE 1:** Available tests using NGS technology

Type of NGS	Method	Performing lab
Targeted NGS, also known as broad-range PCR with NGS	PCR amplification of targeted genes (16S rDNA [bacteria/mycobacteria], *rpoB*, and *hsp65* [mycobacteria] or internal transcribed spacer regions [fungi]) directly from patient specimens (fresh tissue or biopsy, paraffin-embedded tissue block, and body fluid including CSF and synovial fluid) followed by sequencing and analysis using a curated microbial genome database	
	Bacterial, fungal, nontuberculous mycobacterial, and/or *Mycobacterium tuberculosis* complex targets, all of which must be ordered separately based on the suspected pathogen	University of Washington Molecular Diagnosis Microbiology Section, Seattle, WA, USA
	Bacteria (including mycobacteria)	Mayo Clinic Laboratories, Rochester, MN, USA
mNGS, also known as cell-free NGS or liquid biopsy	Untargeted or “shotgun” sequencing of all the cell-free DNA within plasma or CSF specimens and apply bioinformatics to identify the pathogenic organism(s)	
	Plasma	Karius Inc., Redwood City, CA, USA
	CSF	Delve Bio, Inc., San Francisco, CA, USA

The Karius mNGS test uses plasma as the specimen type and is based on the finding that cell-free DNA originating from pathogens circulates freely in the bloodstream, even if the primary infection is located elsewhere. Since the cerebrospinal fluid (CSF) is housed within a protected compartment, the ability of the Karius mNGS test to detect pathogens causing infections localized to the CNS is less consistent. For suspected CNS infections where an agnostic approach is desired, mNGS with CSF as the specimen type (Delve Bio CSF) is preferred.

The main shortcomings of mNGS are the high cost and the potential for polymicrobial results or reporting of possible commensal organisms. To aid in interpretation, the Karius test report quantifies each organism detected as DNA molecules/microliter. Higher concentrations are generally more likely to be clinically relevant but require careful analysis within the context of each patient (6).

NGS is best utilized when there is clear laboratory or strong clinical evidence of infection, but conventional lab tests have failed to produce a diagnosis. To optimize use, many medical centers have developed internal guidance for the use of NGS. Standard of care testing, including cultures, antigen testing, serology, targeted PCR, histopathology, etc., is done first as appropriate for each patient. If standard testing is negative or inconclusive at 48–72 hours and there is a high probability of infection and the patient is expected to receive long-term (≥14 days) antimicrobial therapy, NGS testing can be considered. If a tissue or fluid specimen from the site of infection is available, targeted NGS may be preferred over plasma mNGS. Plasma mNGS can be useful in a patient who is too clinically unstable to undergo invasive specimen collection from the site of infection or when an untargeted approach that detects any pathogen(s) present in the sample is desired. mNGS of CSF is often used when standard tests fail to identify a pathogen in complex cases of suspected infectious central nervous system infection (9, 11).

In many institutions, ordering NGS or mNGS tests is restricted to infectious disease providers and requires microbiology director approval. Investigations have shown a wide range of utility, depending on the study design, definitions used, etc., but many emphasize that diagnostic power and clinical impact are improved when there is rigorous stewardship (2–4, 6, 7, 14).

Phillips et al. (1) reported a case of an infant with retinoblastoma who presented with clinical and radiographic signs of meningitis and infectious intracranial aneurysms. Diagnostic work-up included bacterial, fungal, and mycobacterial cultures of blood and CSF, all of which showed no growth. Very high β−1,3-D-glucan (BDG) levels in CSF and moderate levels in serum as well as negative *Aspergillus* galactomannan led to a working diagnosis of fungal meningitis. Positive cryptococcal antigen titers in CSF and serum complicated the diagnostic picture because BDG is not expected to be positive in *Cryptococcus* infections since the organism is known to produce undetectable low levels of BDG. Because of the uncertainty raised by these results, plasma and CSF samples were sent for mNGS. Plasma cell-free mNGS returned a result of three different organisms with *T. inkin* the only fungus detected and most consistent with clinical suspicion even though present in lowest quantity. *T. inkin* was later detected in CSF using mNGS and metagenomic multiple extended locus typing phylogenetic analysis. Confidence in the *T. inkin* diagnosis raised concern about possible cross-reactivity of the IMMY CrAg cryptococcal antigen lateral flow immunochromatographic assay (LFA).

The package insert for the IMMY CrAg LFA lists cross-reactivity with *Trichosporon beigelli* as a limitation but refers to a manuscript that reports false-positive results for two patients with disseminated fungal infection caused by *Trichosporon asahii* (15, 16). A subsequent publication supports the *T. asahii* cross-reactivity (17). It is known that clinical isolates of *T. beigelli* have a cell wall glucuronoxylomannan antigen that is biochemically similar to the glucuronoxylomannan capsular antigen of *Cryptococcus and* cross-reacts in cryptococcal antigen tests (18). But with advances in DNA sequence analysis technology, *Trichosporon* taxonomy has been progressively revised to remove *T. beigelli* and create several new species (19). Cell wall glucuronoxylomannan is likely a shared cross-reactive antigen across other *Trichosporon* species, but *T. asahii* has been the only species specifically tested in cryptococcal antigen tests prior to publication of the report commented on here.

Since the cryptococcal antigen result in this case seemed to be inconsistent with the final *T. inkin* diagnosis, the authors explored the possibility of cross-reactivity. They tested 0.5 McFarland suspensions of known *T. inkin* isolates from three patients and obtained positive results in the lateral flow cryptococcal antigen test. This supported their speculation and provided an explanation for the inconsistent laboratory results.

Having up-to-date information and knowing the limitations outlined in the manufacturer’s package insert is crucial when troubleshooting unusual test results. By referring to known cross-reactivity, interfering substances, or conditions where the test may not perform as expected, healthcare professionals can identify whether unexpected results stem from known constraints of the test rather than true clinical findings, helping to avoid misinterpretation and guiding appropriate follow-up actions.

This case illustrates that the advantages of mNGS can expand beyond the ability of the test to provide a specific etiologic diagnosis. The case also highlights the importance of open communication between the clinical microbiology laboratory and clinical care teams, especially in complicated cases like this.

I commend the clinical microbiology laboratory at Weill Cornell Medicine, New York City, USA, for working closely with their clinical teams, requesting assistance from mNGS laboratory experts to obtain a species identification for the CSF sample, and taking extra steps to support their suspicion of cross-reactivity, which helped to provide sound rationale for the observed test results and give clinical clarity. Further satisfying was that antifungal treatment was successful in clearing the infection in this very serious case.
